# Admission Serum Sodium as an Early Integrative Marker of Shock Severity in Acute Myocardial Infarction-Complicated Cardiogenic Shock

**DOI:** 10.3390/biomedicines14092086

**Published:** 2026-09-16

**Authors:** Xi Wang, Qianfeng Xiao, Fangyang Huang, Si Wang, Ying Xu, Mao Chen, Xin Wei

**Affiliations:** Department of Cardiology, West China Hospital, Sichuan University, 37 Guoxue Street, Chengdu 610041, China; christina.wangxi@foxmail.com (X.W.); cardiofengzaizai@126.com (Q.X.); fyhuang1989@126.com (F.H.); si.wang@scu.edu.cn (S.W.); xy_123456@wchscu.cn (Y.X.); hmaochen@vip.sina.com (M.C.)

**Keywords:** cardiogenic shock, serum sodium level, hypernatremia, mortality

## Abstract

**Background**: Serum sodium is an important laboratory value in hospitalized patients, but its relationship with mortality in patients with cardiogenic shock complicated by acute myocardial infarction (AMI-CS) has not been evaluated. We assessed the association between admission serum sodium and mortality in AMI-CS. **Methods**: A total of 312 patients with AMI-CS treated at a single center were retrospectively enrolled in this study. The primary endpoint was all-cause mortality. Patients were divided into three groups by tertiles based on admission serum sodium levels. The prognostic value of admission serum sodium levels was evaluated using Kaplan–Meier survival curves and Cox regression. Restricted cubic splines, subgroup analyses, and sensitivity analyses were also performed. Results: Over a follow-up of 4.5 years, 51.9% of enrolled patients died. In unadjusted analyses, higher admission serum sodium was associated with mortality, as well as rates of sepsis, dialysis, and brain injury during hospitalization, and patients with sodium > 141.2 mmol/L had higher short-term mortality; a J-shaped relationship was observed on unadjusted spline analysis. However, this association did not persist after adjustment for shock severity: the fully adjusted hazard ratio per 1 mmol/L was 1.02 (95% CI 0.98–1.06, *p* = 0.46) for long-term and 1.03 (95% CI 0.98–1.07, *p* = 0.28) for 30-day mortality, and the spline association was no longer significant (*p* for overall association = 0.40; *p* for nonlinearity = 0.33). Adding severity variables one at a time showed that the association was abolished specifically by arterial lactate. **Conclusions**: Higher admission serum sodium is associated with mortality in AMI-CS, but this association is largely explained by concurrent shock severity, particularly hyperlactatemia. Admission sodium may serve as an early, readily available integrative marker of shock severity for risk stratification, but whether sodium-directed treatment strategies affect outcomes requires prospective study.

## 1. Introduction

Sodium holds a pivotal position within the human body, serving as the principal extracellular cation and playing a fundamental role in modulating cell volume. The normal reference range for serum sodium level generally lies between 135.0 mmol/L and 145.0 mmol/L [1,2]. Both hyponatremia and hypernatremia pose detrimental effects. Research has demonstrated that sodium imbalance represents a prevalent problem among hospitalized patients, with approximately 30% to 40% being affected. In the context of critically ill patients, the serum sodium level can potentially serve as an indicator of the disease state [3,4]. Roughly 20% of such patients encounter hyponatremia, which impacts 35% of hospitalized patients and 11.2% of those in the intensive care unit (ICU) [2,5]. Hypernatremia, albeit less common, occurs at a rate of 2–6% in ICU patients [6]. It has been established that hypernatremia serves as an independent risk factor for augmented in-hospital and short-term mortality in non-cardiovascular critically ill patients in the ICU [2,3,6]. Analogously, hyponatremia has been linked to cardiovascular events and mortality in patients afflicted with heart failure [7,8,9,10].

Cardiogenic shock (CS) is a primary cardiac disorder leading to hypotension and signs of organ hypoperfusion, occurring in the setting of normovolemia or hypervolemia. Epidemiological data regarding CS indicates that approximately 60–80% of cases are attributed to acute myocardial infarction (AMI). The thirty-day mortality rate among patients with CS caused by AMI is around 40%, and the 1-year mortality rate nears 50% [11,12]. Consequently, the identification of prognostic markers capable of guiding early risk stratification and enhancing outcomes persists as a significant clinical conundrum for patients with cardiogenic shock following acute myocardial infarction (AMI -CS). Notably, there is a paucity of knowledge concerning the incidence and prognostic significance of dysnatremia in critically ill patients with AMI-CS admitted to the cardiac care unit (CCU). In this study, our objective is to investigate the correlation between admission serum sodium concentration and mortality rates among AMI-CS patients.

## 2. Methods

### 2.1. Study Population and Definitions

**Figure 1:**
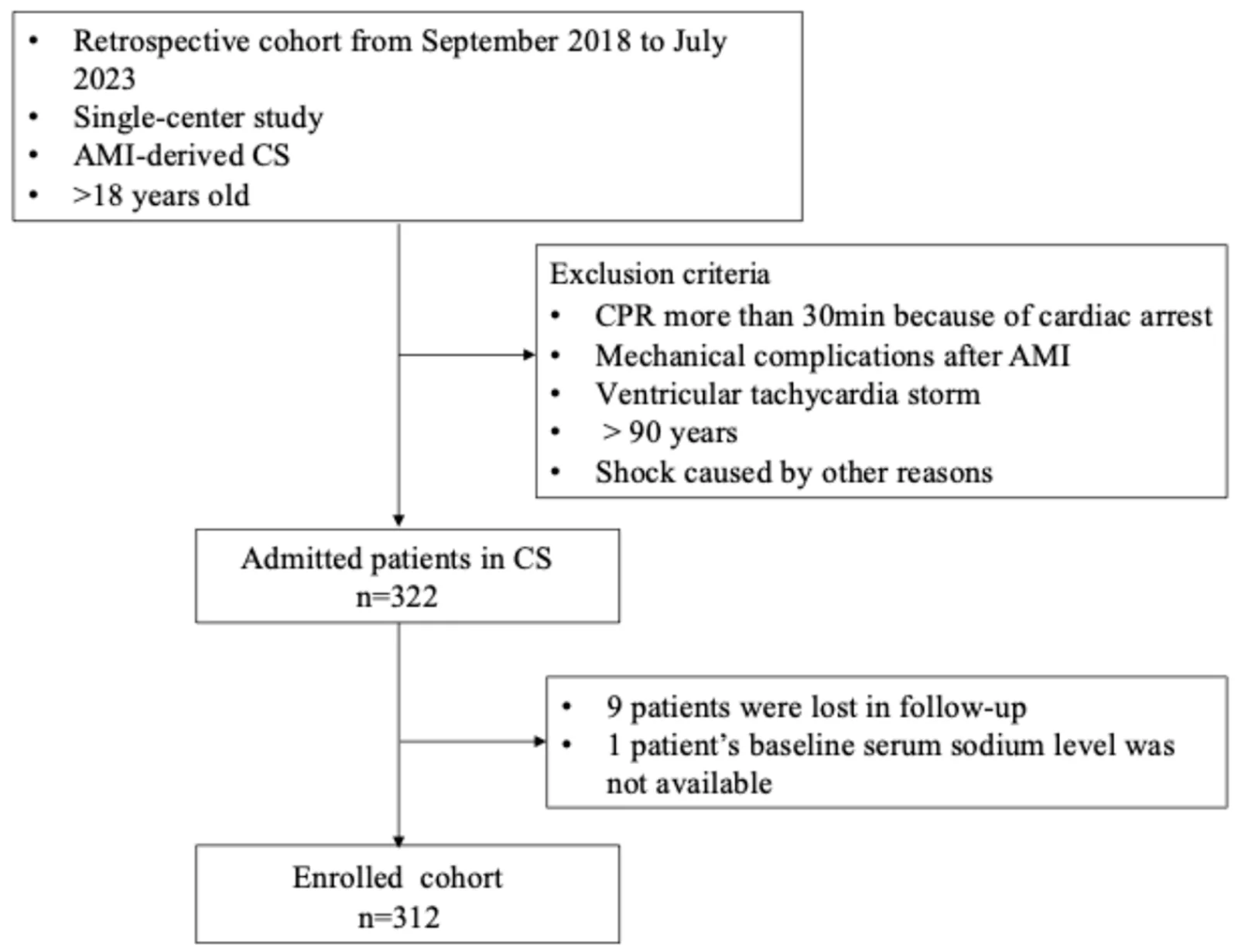
Study design. Abbreviations: AMI = acute myocardial infarction. CS = cardiogenic shock. CPR = cardiopulmonary resuscitation.

This single-center retrospective cohort study included patients diagnosed with AMI-CS who received medical treatment from September 2018 to July 2023 at the West China Hospital of Sichuan University, renowned as the largest tertiary care hospital in the western region of China (Figure 1). Patients involved met the following inclusion criteria: (a) AMI diagnosed per the fourth universal definition of myocardial infarction [13]: Acute myocardial injury with clinical evidence of acute myocardial ischemia (myocardial ischemic symptoms, new ischemic ECG changes, pathological Q waves, imaging evidence of new viable myocardium loss or regional wall motion abnormality consistent with ischemic etiology, or coronary thrombus identified by angiography/autopsy), and cardiac troponin elevation with at least one value >99th percentile upper reference limit. (b) Cardiogenic shock: a primary cardiac disorder leading to hypotension (systolic blood pressure < 90 mmHg, or vasopressors required to maintain systolic blood pressure ≥ 90 mmHg) and organ hypoperfusion signs (increased arterial lactate > 2 mmol/L, oliguria, altered mental status, cold and clammy skin/extremities) in the state of normovolemia or hypervolemia [11]. (c) Cardiogenic shock stages B to E per the Society for Cardiovascular Angiography and Interventions (SCAI) criteria [11]. The exclusion criteria were as follows: (a) Cardiopulmonary resuscitation (CPR) time of >30 min due to pre-admission cardiac arrest. (b) CS due to mechanical complications post-AMI. (c) CS due to ventricular tachycardia storm. (d) Shock from other etiologies (e.g., septic/hemorrhagic shock), and (e) age > 90 years.

### 2.2. Baseline Data Collection

Demographic details, vital signs, and medical history, along with information regarding mechanically supported therapies (such as mechanical circulatory support and ventilation data) and the specific AMI type were collected from the hospital medical records. Admission serum sodium was defined as the value from the first venous blood sample drawn after arrival at our institution, measured in the emergency department or CCU at the time of the initial diagnostic work-up; when more than one measurement was available during the admission period, only this first post-arrival value was used, so as to reflect sodium status as close as possible to presentation and before subsequent in-hospital fluid administration, diuretics, or contrast exposure. Baseline blood gas analysis and biochemical test results were obtained at the same time as the admission venous blood draw. The coronary angiography procedural data were sourced from corresponding images. Cardiac function, including left ventricular ejection fraction (LVEF), was determined by echocardiography reports at admission. Cardiogenic shock scores (intra-aortic balloon pump in cardiogenic shock II, IABP SHOCK II) were calculated according to the baseline data [14]. These scores were evaluated with age, history of stroke, serum glucose, creatine, arterial lactate, and thrombolysis in myocardial infarction (TIMI) flow grade 3 achieved after percutaneous coronary intervention (PCI).

### 2.3. Study Endpoints

The primary endpoint was all-cause death from hospital admission to follow-up. Secondary endpoints were the occurrence of sepsis, the need for dialysis and the incidence of brain injury. Follow-up information was obtained through telephone conversations, review of medical charts, and outpatient consultations. The integrity and accuracy of all data were verified and supported by the official hospital records, ensuring the reliability and validity of the information collected for the study. For the small number of patients who died on the day of admission and therefore had a recorded follow-up time of zero, survival time was derived from the CCU length of stay (hours divided by 24) to permit time-to-event modeling.

### 2.4. Ethics and Patients’ Consent

This study was approved by the Institutional Review Board of West China Hospital, Sichuan University (Chengdu, China; approval number: 2021–1770) on 13 January 2022.

### 2.5. Statistical Analysis

Patients included in this study were stratified into three groups based on admission serum sodium tertiles. The Kolmogorov–Smirnov test was employed to assess data distribution. Variables were presented as the means and medians, or frequencies and percentages, together with their corresponding ranges. The analysis of variance or the Kruskal–Wallis test was utilized across groups for continuous variables, and chi-squared or Fisher’s exact test was applied to make comparisons of the qualitative variables among the groups. Survival rates were depicted in the form of Kaplan–Meier plots, and intergroup differences were analyzed with the log-rank test. Associations between variables and endpoints were first evaluated via univariate Cox regression. Covariates for the multivariable models were prespecified on clinical grounds, rather than selected solely by univariable significance. The dose–response relationship between admission serum sodium and mortality was examined using restricted cubic splines within Cox models, with four knots placed at the 5th, 35th, 65th, and 95th percentiles of the sodium distribution and the cohort median as the reference; splines were fitted both without adjustment and with adjustment for the shock severity covariates—arterial lactate, out-of-hospital cardiac arrest (OHCA), mechanical ventilation, and mechanical circulatory support (MCS)—and *p* values for the overall association and for nonlinearity were obtained from Wald tests of all spline terms and of nonlinear terms, respectively. The proportional hazards assumption was assessed using scaled Schoenfeld residuals (global and covariate-specific tests). Where non-proportionality was detected, sensitivity analyses were performed, allowing a period-specific covariate effect (before vs. after day 30) and a 30-day landmark analysis, and hazard ratios were interpreted as time-averaged effects over the relevant follow-up period. To investigate the potential heterogeneity in the impact of admission serum sodium levels on all-cause mortality, subgroup analyses were carried out. The subgroups comprised age categories (with a cutoff of 65 years), gender, OHCA, stroke history, diabetes status, serum creatinine levels (with a cutoff of 132.6 µmol/L), ventilation status, and MCS. These subgroup analyses were regarded as exploratory and hypothesis-generating, and no correction for multiple comparisons was applied. A two-sided *p* value < 0.05 was defined as statistically significant, while a *p* value < 0.1 was considered significant for interaction tests. All analyses were performed using Stata (version 17.0, StataCorp LLC., College Station, TX, USA), R software (version 4.5.3, Free Software Foundation, Inc., Boston, MA, USA) and GraphPad Prism (version 9, GraphPad Software, LLC., Boston, MA, USA).

## 3. Results

**Figure 2:**
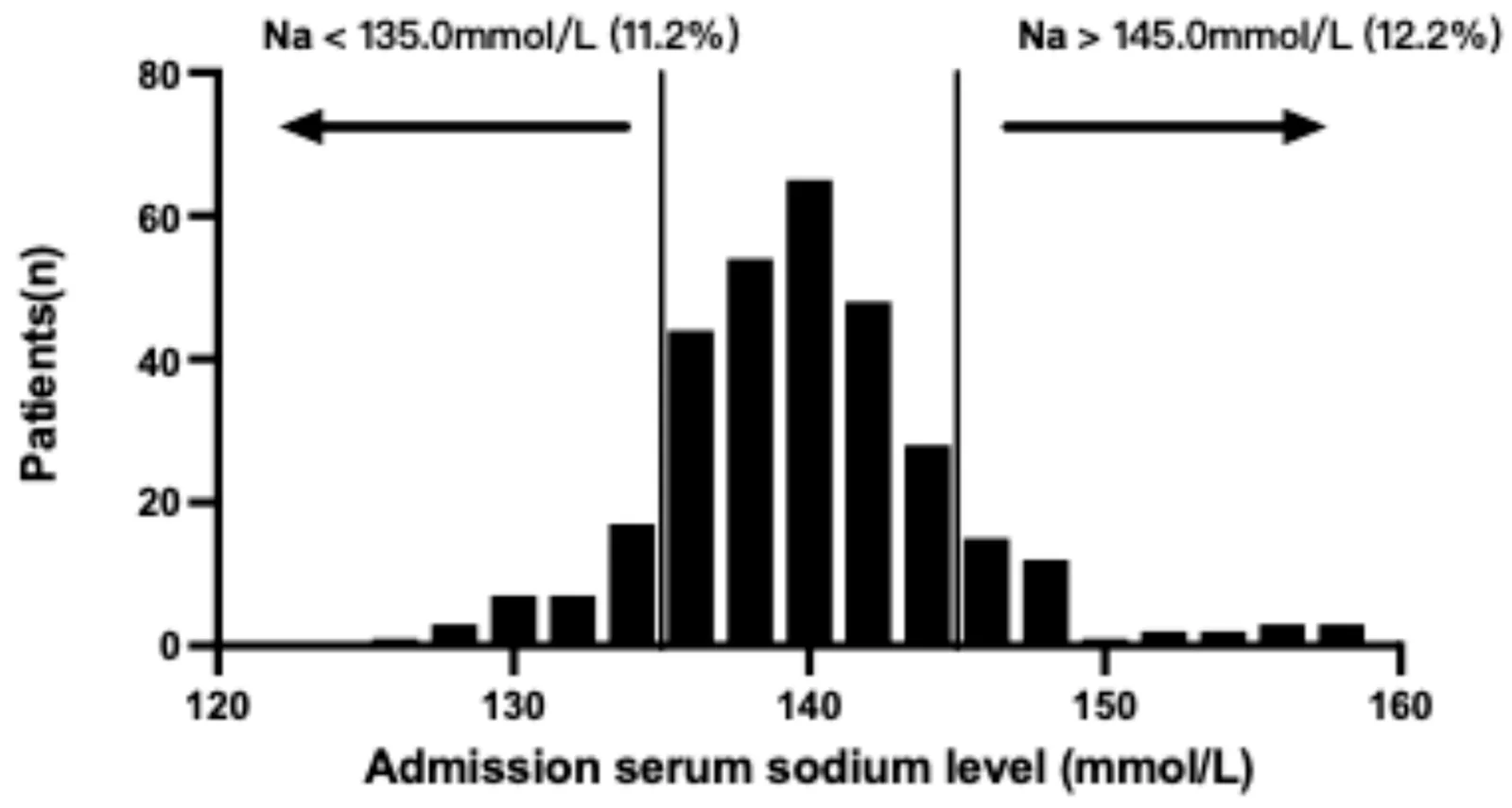
Distribution of admission serum sodium in AMI-CS patients. Abbreviation: AMI-CS = cardiogenic shock followed by acute myocardial infarction.

A total of 312 AMI-CS patients was finally included in this study (Figure 1). The mean serum sodium level was 140.0 ± 5.0 mmol/L and the histogram depicted a broad dispersion of the admission sodium values (Figure 2). Hyponatremia (<135 mmol/L) and hypernatremia (>145 mmol/L) were present in 11.2% and 12.2% of patients, respectively. In accordance with the admission serum sodium level, the patients were stratified into three distinct groups using tertiles: those with Na < 137.9 mmol/L, 138.0 ≤ Na ≤ 141.2 mmol/L, and Na > 141.2 mmol/L. Baseline characteristics of each group are presented in Table 1. The mean age of the patients was 67.6 years, with a total of 238 (76.0%) patients being male. Notably, there were no differences in age and sex distribution across groups. Upon admission, there were no discernible differences in systolic blood pressure (SBP), mean arterial pressure (MAP), or heart rate among patients. Moreover, no perceptible discrepancies were observed in the occurrence rates of comorbidities such as hypertension, coronary artery disease (CAD), stroke, chronic kidney disease (CKD) and peripheral arterial disease (PAD) across the studied groups. Importantly, patients in the highest sodium group were more severely ill across multiple markers of shock severity: they had a higher frequency of OHCA (29.8% vs. 19.4% vs. 9.5%), and higher arterial lactate (6.77 vs. 4.10 vs. 3.85 mmol/L), serum creatinine (157.65 vs. 148.17 vs. 148.05 µmol/L), and troponin T. They also more often required mechanical ventilation (86.5% vs. 73.8% vs. 67.6%) and had more advanced SCAI stages and higher IABP-SHOCK II scores (all *p* < 0.05; Table 1).

Overall, 73.4% of the patients were diagnosed with ST-segment elevation myocardial infarction (STEMI), among whom 49.4% were specifically categorized as having anterior wall infarction. The ratios of patients who received coronary angiography, revascularization, coronary lesions and TIMI flow grade after PCI did not display any pronounced differences across the groups. Moreover, 51.3% of the patients were provided with mechanical circulatory support, and the Na > 141.2 mmol/L group demonstrated a higher prevalence of extracorporeal membrane oxygenation (ECMO) implantation. Nevertheless, LVEF showed no significant intergroup differences.

The median follow-up duration was 291 days. At the longest follow up—4.5 years, a total of 162 individuals (51.9%) died, and among them, 129 cases occurred within the initial 30 days of the follow-up period. Additionally, 140 (44.9%) patients died due to cardiac-related causes (Table 2). The Na > 141.2 mmol/L group exhibited greater all-cause mortality in comparison to the other two groups (62.5% vs. 44.7% vs. 48.6%, *p* = 0.026). The incidence rates of sepsis, dialysis, and brain injury in the hospital in the total cohort were 12.2%, 14.7%, and 4.2% correspondingly. Remarkably, the occurrences of sepsis, dialysis, and brain injury were more pronounced in the Na > 141.2 mmol/L group, with respective rates of 15.4%, 18.3%, and 8.7% compared to the other groups (Table 2).

**Figure 3:**
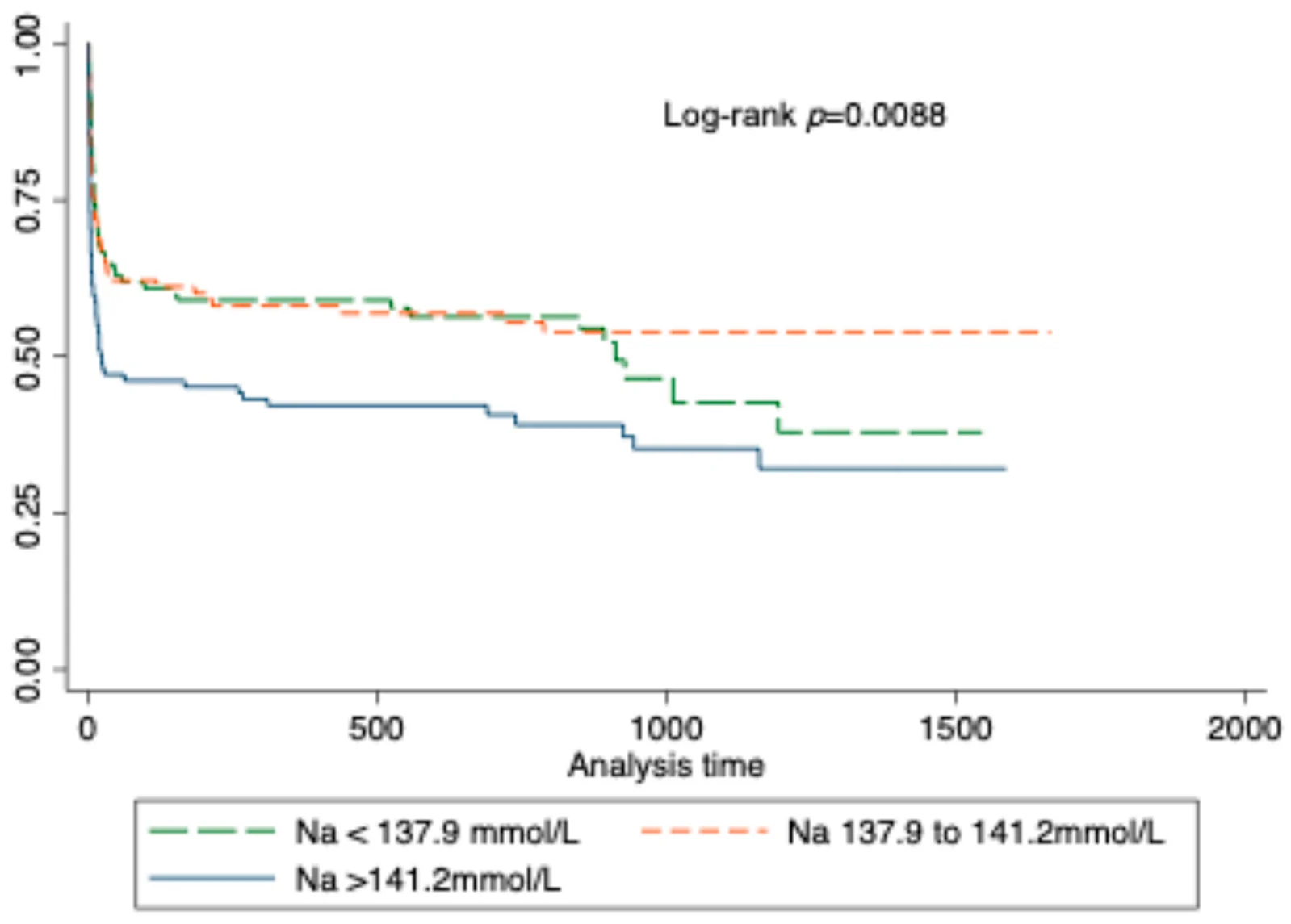
Kaplan–Meier survival curve grouped by admission serum sodium levels in AMI-CS patients. Abbreviation: AMI-CS = cardiogenic shock followed by acute myocardial infarction.

**Figure 4:**
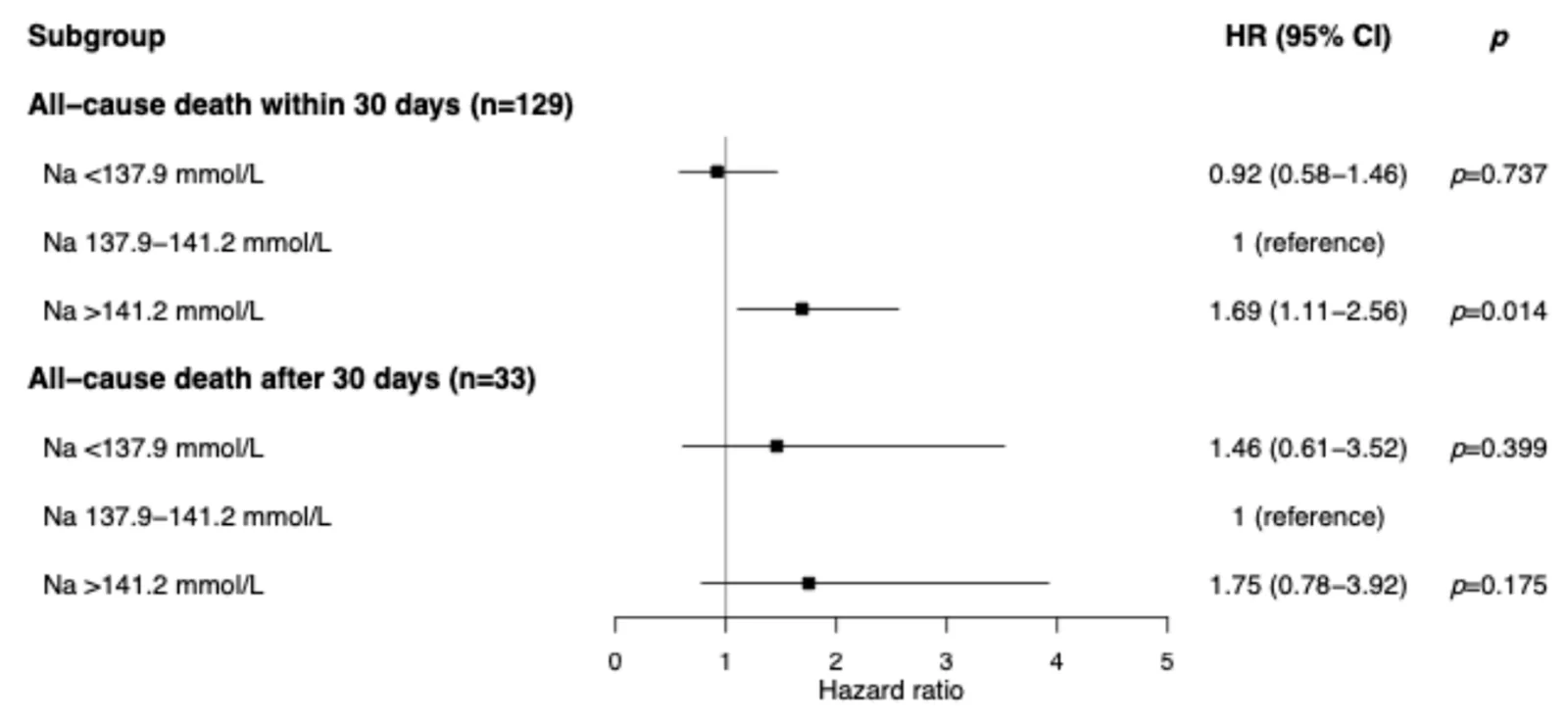
Unadjusted hazard ratios for all-cause deaths within 30 days and after 30 days, grouped by admission serum sodium levels.

The Kaplan–Meier survival curve (Figure 3) revealed that, in contrast to the other groups, the Na > 141.2 mmol/L group exhibited a significantly elevated long-term (4.5 years) mortality rate (*p* = 0.0088). In unadjusted analyses, higher admission sodium was associated with mortality both as tertiles and as a continuous variable (crude HR per 1 mmol/L 1.07, 95% CI 1.03–1.11; Figure 4, Table 3). However, this association did not persist after adjustment for shock severity. In multivariate Cox models (Table 3), the crude association of admission sodium with long-term mortality was largely unchanged after adjustment for demographic and baseline clinical variables (HR 1.05, 95% CI 1.02–1.09) but was abolished after further adjustment for arterial lactate, OHCA, mechanical ventilation, and mechanical circulatory support (HR 1.02, 95% CI 0.98–1.06, *p* = 0.46). The same pattern was observed for 30-day mortality (fully adjusted HR 1.03, 95% CI 0.98–1.07, *p* = 0.28).

**Figure 5:**
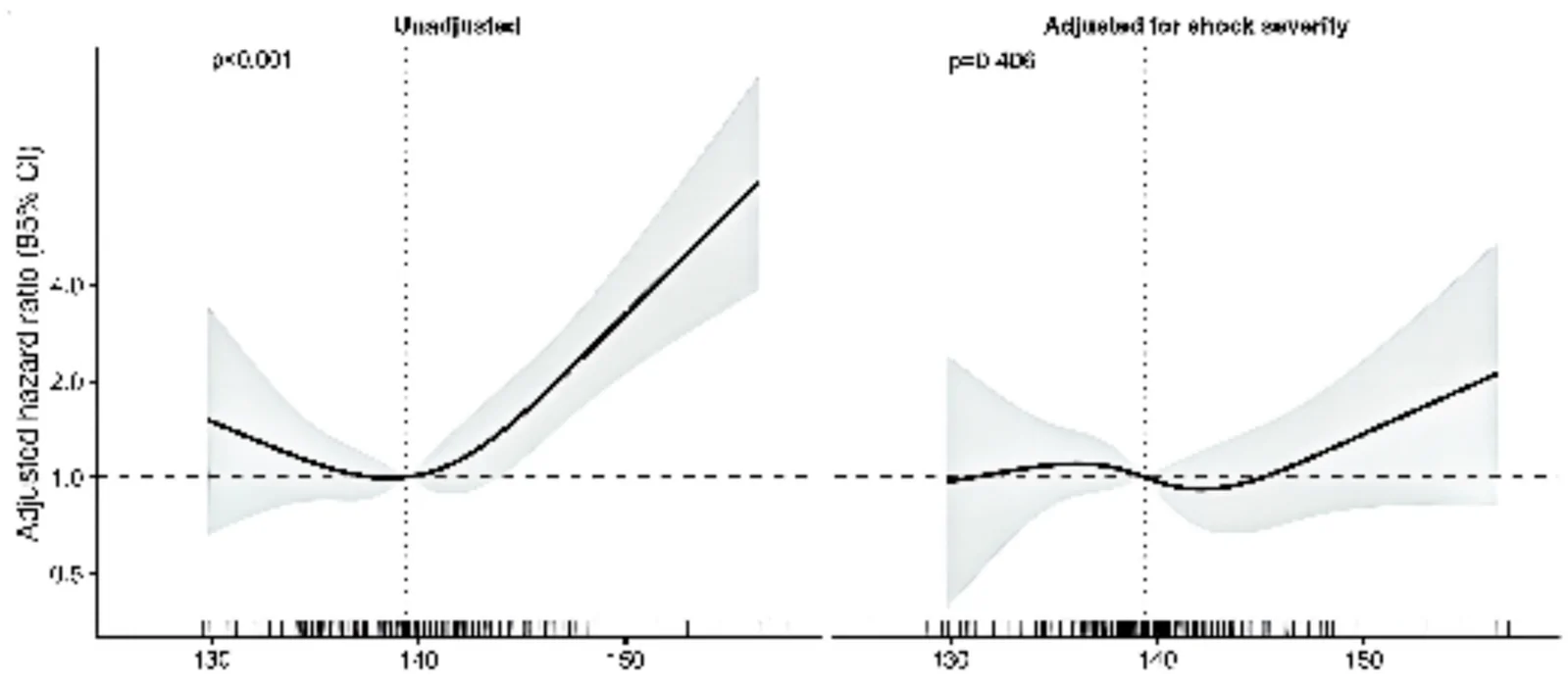
Restricted cubic spline curves for the association between admission serum sodium and long-term all-cause mortality, before (**left**) and after (**right**) adjustment for cardiogenic shock severity (arterial lactate, out-of-hospital cardiac arrest, mechanical ventilation, and mechanical circulatory support). Splines used four knots placed at the 5th, 35th, 65th, and 95th percentiles of the sodium distribution (132.6, 138.1, 140.9, and 148.0 mmol/L); the cohort median (139.4 mmol/L) served as the reference (dashed line). Solid lines denote hazard ratios, and shaded areas 95% confidence intervals.

Restricted cubic spline analysis was concordant. Before adjustment, admission sodium showed a J-shaped relationship with mortality (*p* for overall association < 0.001; *p* for nonlinearity = 0.003); after adjustment for shock severity variables, neither the overall nor the nonlinear association remained significant (*p* for overall association = 0.40; *p* for nonlinearity = 0.33, Figure 5). In analyses adding one severity variable at a time, the sodium association was attenuated only by arterial lactate and was largely unchanged by OHCA, ventilation, or the IABP-SHOCK II score (Table 4). These stepwise analyses suggest that lactate, rather than other binary markers of illness severity, is the key covariate that attenuates the sodium signal. These findings were robust across sensitivity analyses (Supplementary Table S1).

**Figure 6:**
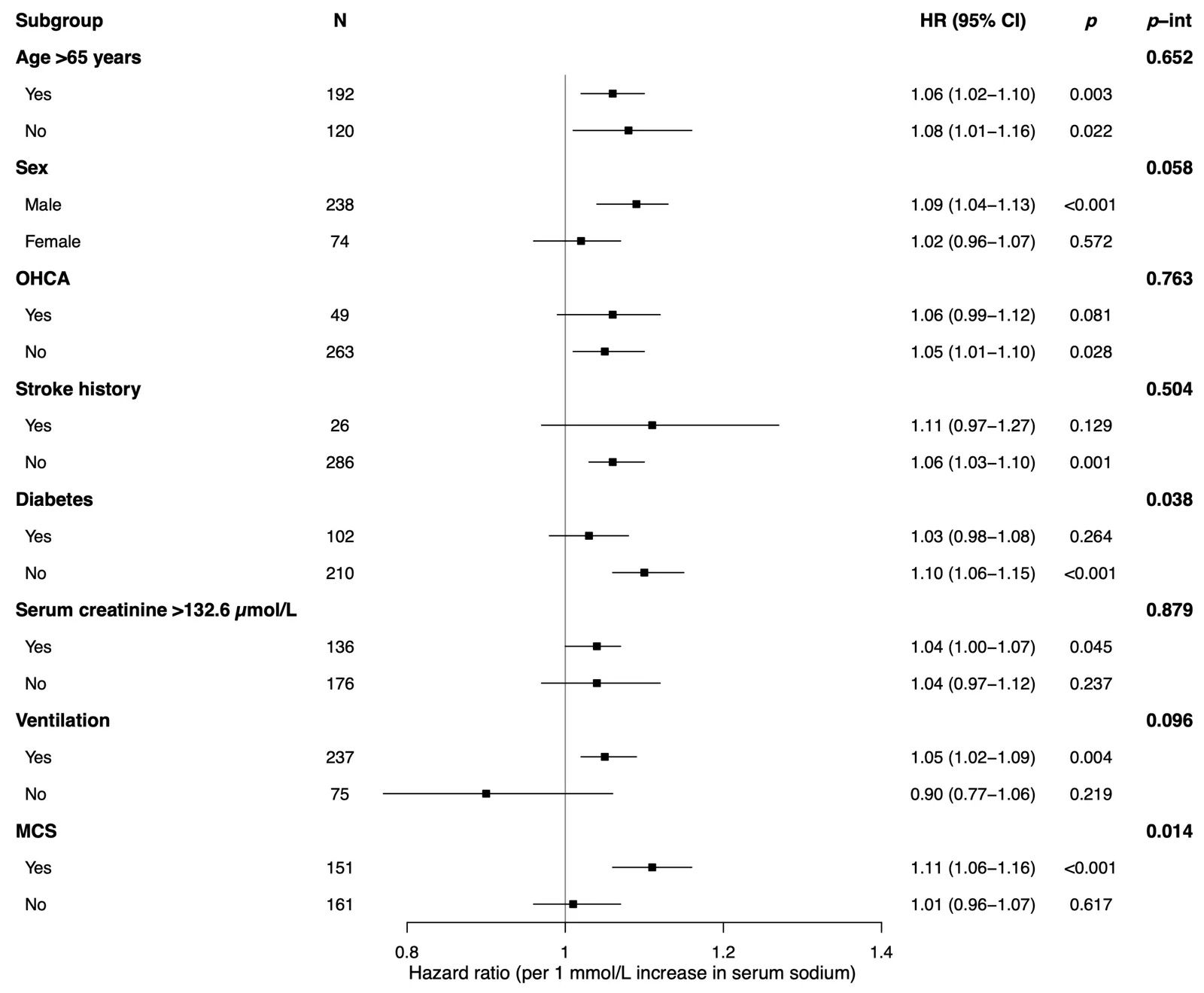
Exploratory subgroup analysis of the association between admission serum sodium (per 1 mmol/L increase) and long-term mortality across prespecified clinical subgroups. Hazard ratios are unadjusted and shown with *p* values for interaction; binary subgroups are defined as yes versus no.

In exploratory subgroup analyses (Figure 6), the unadjusted association between admission sodium and mortality appeared numerically stronger in men, in patients without diabetes, and in those receiving mechanical circulatory support. However, these analyses were hypothesis-generating, no correction for multiple comparisons was applied, and the *p* values for interaction (all between 0.05 and 0.10 for sex, and 0.04 and 0.018 for diabetes and MCS) should be interpreted cautiously; the female subgroup was small (*n* = 74).

## 4. Discussion

Our research is dedicated to exploring the relationship between serum sodium concentration and mortality in AMI-CS patients. We found that higher admission sodium was associated with mortality in unadjusted analyses, but that this association did not persist after adjustment for markers of cardiogenic shock severity, particularly arterial lactate. Rather than being an independent predictor, admission sodium appears to be an early, readily available integrative marker that reflects the severity of shock and its associated resuscitation and organ dysfunction. To our knowledge, this is the first study to examine admission sodium specifically in AMI-CS, and it highlights the importance of accounting for shock severity when interpreting this common laboratory value.

Serum sodium concentration is of paramount importance in upholding the water and electrolyte balance within the human body. Dysnatremia, which is prevalent in electrolyte disturbances among hospitalized patients, typically originates from imbalances in the intake and loss of electrolyte-free water [4]. Roughly 20–30% of heart failure (HF) patients exhibit dysnatremia upon admission, prompting the European Society of Cardiology heart failure guideline to recommend in-hospital monitoring of serum sodium level [7,15]. In previous reports, the rates of hyponatremia and hypernatremia were recorded as 16.37% and 8.16%, respectively, in a cohort of 4760 heart failure patients [6,7]. Nevertheless, no studies have explored serum sodium concentrations in CS patients, particularly those caused by AMI. In our AMI-CS cohort, hyponatremia (<135 mmol/L) occurred in 11.2% and hypernatremia (>145 mmol/L) in 12.2% of patients. The underlying causes of hypernatremia in these patients have yet to be elucidated. It is hypothesized that increased activity in the sympathetic nervous and renin–angiotensin–aldosterone systems in AMI-CS patients leads to elevated levels of aldosterone and subsequent sodium retention. Renal insufficiency may precipitate significant water loss and cause hypernatremia [6]. Additionally, critically ill patients, who frequently necessitate ventilation or circulatory support, encounter challenges in accessing water, thereby further heightening their susceptibility to hypernatremia.

Prior studies have linked both hyponatremia and hypernatremia to higher mortality in heart failure, acute myocardial infarction, and general ICU populations [3,6,8,9,10,15]. Our unadjusted findings are consistent with these reports. The key observation of the present study, however, is that in AMI-CS, the association between higher admission sodium and death is largely explained by concurrent shock severity. In our data, once arterial lactate was included in the model, admission sodium no longer carried independent prognostic information, suggesting that high sodium might be an upstream indicator of elevated lactate level and the subsequent severe shock. The potential mechanism behind this is that hypernatremia might exacerbate peripheral insulin resistance and hyperglycemia, thus impairing hepatic gluconeogenesis and lactate clearance [16,17]. Other mechanisms which plausibly link sodium with shock severity include accompanying neurological injury [9,18] and association with negative inotropic effects on the myocardium [19,20], as well as typically signifying water loss and hypovolemia.

Our subgroup analyses were exploratory. The association between admission sodium and mortality appeared numerically stronger in men, in patients without diabetes, and in those receiving MCS, but the interaction tests were weak, multiple subgroups were examined without correction, and the female subgroup was small. Any apparent differences—for example, a weaker association in diabetic patients, in whom osmotic effects of hyperglycemia may raise sodium without reflecting true severity [16], or a stronger association in patients supported with IABP or ECMO, who are predisposed to infection and sepsis [21,22]—should therefore be regarded as hypothesis-generating and not as evidence of subgroup-specific effects.

Clinically, our findings suggest that admission sodium may be useful as a simple, rapidly available marker for early risk stratification in AMI-CS, but that its prognostic value is largely a reflection of downstream lactate level and overall shock severity, rather than an independent target. Accordingly, we do not propose a specific “optimal” sodium range, and our data do not support sodium-directed interventions. Whether measures that influence sodium—such as fluid strategy, avoidance of unnecessary sodium loading, use of sodium glucose cotransporter 2 (SGLT2) inhibitors, or renal replacement therapy—affect outcomes in AMI-CS cannot be inferred from this observational association and would require prospective evaluation.

This study had several limitations. Firstly, this was a single-center, observational study with a relatively small sample size, which might have rendered it underpowered to detect certain differences. Secondly, although we adjusted for established markers of shock severity, residual and unmeasured confounding cannot be excluded; conversely, several of these markers are closely related to sodium, so the adjusted estimates may be conservative. Thirdly, although admission sodium was taken from the first sample obtained after arrival, some patients may have received pre-hospital or referring-hospital fluids, resuscitation (including sodium bicarbonate during CPR), diuretics, or contrast before this measurement; transfer status was not recorded, so treatment-related contributions to admission sodium cannot be fully excluded, particularly after OHCA. Finally, subgroup and nonlinearity analyses were exploratory. Admission sodium should therefore be interpreted as an early integrative marker, rather than an isolated pathophysiological variable, and prospective multicenter studies are needed to determine whether sodium-directed strategies have any effect on outcomes in AMI-CS.

## 5. Conclusions

In this AMI-CS cohort, higher admission serum sodium was associated with all-cause mortality in unadjusted analyses, but the association did not remain after adjustment for markers of cardiogenic shock severity, particularly arterial lactate. Admission serum sodium therefore appears to be an early, readily available integrative marker of shock severity, rather than an independent predictor, and may aid rapid risk stratification. Whether sodium-directed strategies influence outcomes in AMI-CS should be examined in prospective, multicenter studies.

## Figures and Tables

**Table 1:** Baseline and clinical characteristics of patients stratified by admission serum sodium levels in AMI-CS patients.

	Total (*n* = 312)	<137.9 (*n* = 105)	138.0–141.2 (*n* = 103)	>141.2 (*n* = 104)	*p* Value
**Demographic data**					
Age, years	67.6 ± 12.9	68.0 ± 12.5	65.2 ± 14.4	69.4 ± 11.4	0.059
Male (%)	238/312 (76.0)	82/105 (78.1)	80/103 (77.7)	76/104 (73.1)	0.641
BMI, kg/m^2^	23.5 ± 3.5	23.2 ± 3.7	23.8 ± 3.7	23.5 ± 3.0	0.064
SBP, mmHg	92.6 ± 15.1	92.8 ± 15.1	94.4 ± 16.2	90.6 ± 14.0	0.321
MAP, mmHg	72.0 ± 11.8	72.9 ± 12.0	72.9 ± 12.5	70.1 ± 10.6	0.229
Heart rate, bpm	97.7 ± 22.5	94.8 ± 20.1	100.6 ± 23.5	97.9 ± 23.5	0.185
OHCA (%)	61/312 (19.6)	10/105 (9.5)	20/103 (19.4)	31/104 (29.8)	0.001
**Cardiovascular risk factors/CVD**					
Smoking (%)	150/312 (48.1)	53/105 (50.5)	52/103 (50.5)	45/104 (43.3)	0.486
Arterial hypertension (%)	149/312 (47.8)	56/105 (53.3)	41/103 (39.8)	52/104 (50.0)	0.127
Diabetes mellitus (%)	102/312 (32.3)	44/105 (41.9)	33/103 (33.0)	24/104 (23.1)	0.015
History of CAD (%)	61/312 (19.6)	17/105 (16.2)	20/103 (19.4)	24/104 (23.1)	0.455
History of stroke (%)	26/312 (8.3)	5/105 (4.8)	7/103 (6.8)	14/104 (13.5)	0.059
Dyslipidemia (%)	51/312 (16.7)	17/105 (16.2)	21/103 (20.4)	14/104 (13.5)	0.404
Known PAD (%)	8/312 (2.6)	2/105 (1.9)	3/103 (2.9)	3/104 (2.9)	0.871
CKD (%)	24/312 (7.7)	8/105 (7.6)	10/103 (9.7)	6/104 (5.8)	0.568
**Laboratory results**					
Serum sodium, mmol/L	140.0 ± 5.0	135.1 ± 2.5	139.7 ± 0.9	145.2 ± 4.0	<0.001
Arterial lactate, mmol/L	4.91 ± 4.20	3.85 ± 3.59	4.10 ± 3.29	6.77 ± 4.92	<0.001
Glucose, mmol/L	12.20 ± 6.28	12.56 ± 6.54	11.82 ± 6.44	12.20 ± 5.87	0.505
Serum creatinine, µmol/L	149.29 ± 112.45	148.05 ± 117.19	148.17 ± 126.88	157.65 ± 76.43	<0.001
TnT, ng/L	10,000	8662	8274	10,000	0.026
NT-proBNP, ng/L	9628	9343	8808	10,738	0.518
STEMI (%)	229/312 (73.4)	77/105 (73.3)	70/103 (68.0)	82/104 (78.9)	0.208
Anterior STEMI (%)	154/312 (49.4)	52/105 (49.5)	47/103 (45.6)	55/104 (52.9)	0.580
Ventilation (%)	237/312 (76.0)	71/105 (67.6)	76/103 (73.8)	90/104 (86.5)	0.005
CAG (%)	284/312 (91.0)	97/105 (92.4)	92/103 (89.3)	95/104 (91.4)	0.735
**Coronary lesions**					
Mono-vessel disease (%)	79/283 (27.9)	25/97 (25.8)	27/92 (29.3)	27/94 (28.7)	0.219
Bi-vessel disease (%)	81/283 (28.6)	34/97 (35.1)	18/92 (19.6)	29/94 (30.9)	
Multi-vessel disease (%)	123/283 (43.5)	38/97 (39.2)	47/92 (51.1)	38/94 (40.4)	
Revascularization (%)	259/312 (83.0)	87/105 (82.9)	83/103 (80.6)	89/104 (85.6)	0.632
Complete revascularization (%)	128/312 (41.0)	41/105 (39.1)	40/103 (38.8)	47/104 (45.2)	0.571
TIMI flow grade = 3 after PCI (%)	238/283 (84.1)	78/97 (80.4)	81/92 (88.0)	79/94 (84.0)	0.424
IABP (%)	151/312 (48.4)	45/105 (42.9)	49/103 (47.6)	57/104 (54.8)	0.220
ECMO (%)	9/312 (2.9)	2/105 (1.9)	0/103 (0.0)	7/104 (6.7)	0.012
**SCAI shock stage**					
B (%)	88/312 (28.2)	38/105 (36.2)	31/103 (30.1)	19/104 (18.3)	<0.001
C (%)	148/312 (47.4)	52/105 (49.5)	56/103 (54.4)	40/104 (38.5)	
D (%)	58/312 (18.6)	12/105 (11.4)	11/103 (10.7)	35/104 (33.7)	
E (%)	18/312 (5.8)	3/105 (2.9)	5/103 (4.9)	10/104 (9.6)	
**IABPSHOCK II score**					
0–2 (%)	194/312 (62.1)	78/105 (74.3)	73/103 (70.9)	43/104 (41.3)	<0.001
3–4 (%)	88/312 (28.2)	18/105 (17.1)	23/103 (22.3)	47/104 (45.2)	
5–9 (%)	30/312 (9.7)	9/105 (8.6)	7/103 (6.8)	14/104 (13.5)	
LVEF, %	40.4 ± 11.3	39.5 ± 11.2	41.4 ± 10.2	40.3 ± 12.4	0.156

Abbreviations: AMI-CS = cardiogenic shock followed by acute myocardial infarction. AMI = acute myocardial infarction. CS = cardiogenic shock. BMI = body mass index. SBP = systolic blood pressure. MAP = mean arterial pressure. bpm = beats per minute. OHCA = out of hospital cardiac arrest. CVD = coronary vascular disease. CAD= coronary artery disease. PAD= peripheral artery disease. CKD = chronic kidney disease. TnT = troponin T. STEMI = st-segment elevation myocardial infarction. CAG = coronary angiography. TIMI = thrombolysis in myocardial infarction. PCI = percutaneous coronary intervention. IABP = intra-aortic balloon pump. ECMO = extracorporeal membrane oxygenation. SCAI = Society for Cardiovascular Angiography and Interventions. IABPSHOCK = intra-aortic balloon pump in cardiogenic shock. LVEF = left ventricular ejection fraction.

**Table 2:** The outcomes of the AMI-CS patients grouped by admission serum sodium levels.

	Admission Serum Sodium Level, mmol/L				
	Total (*n* = 312)	<137.9 (*n* = 105)	138.0–141.2 (*n* = 103)	>141.2 (*n* = 104)	*p* Value
Death (%)	162/312 (51.9)	51/105 (48.6)	46/103 (44.7)	65/104 (62.5)	0.026
Cardiac death (%)	140/312 (44.9)	39/105 (37.2)	45/103 (43.7)	56/104 (53.8)	0.009
Non-cardiac death (%)	22/312 (7)	12/105 (11.5)	1/103 (1.0)	9/104 (8.7)	0.01
Sepsis in hospital (%)	38/312 (12.2)	12/105 (11.4)	10/103 (9.7)	16/104 (15.4)	0.440
Dialysis in hospital (%)	46/312 (14.7)	14/105 (13.3)	13/103 (12.6)	19/104 (18.3)	0.458
Brain injury (%)	13/312 (4.2)	1/105 (0.9)	3/103 (2.9)	9/104 (8.7)	0.015

Abbreviation: AMI-CS = cardiogenic shock followed by acute myocardial infarction.

**Table 3:** Univariable and multivariable Cox analyses in mortality of the AMI-CS patients.

Variable	Long-Term Mortality				30-Day Mortality			
	Univariable		Multivariable		Univariable		Multivariable	
	HR (95% CI)	*p*	HR (95% CI)	*p*	HR (95% CI)	*p*	HR (95% CI)	*p*
Serum sodium (per 1 mmol/L)	1.070 (1.029–1.112)	<0.001	1.016 (0.975–1.058)	0.459	1.087 (1.043–1.132)	<0.001	1.025 (0.980–1.072)	0.282
Age (per 1 year)	1.032 (1.016–1.048)	<0.001	1.036 (1.019–1.054)	<0.001	1.040 (1.021–1.059)	<0.001	1.046 (1.025–1.068)	<0.001
SBP (per 1 mmHg)	0.980 (0.967–0.993)	0.002	0.981 (0.969–0.994)	0.005	0.978 (0.963–0.993)	0.004	0.983 (0.968–0.998)	0.031
Heart rate (per 1 bpm)	1.016 (1.008–1.024)	<0.001	1.011 (1.003–1.019)	0.007	1.017 (1.008–1.026)	<0.001	1.012 (1.002–1.021)	0.016
Glucose (per 1 mmol/L)	1.049 (1.023–1.075)	<0.001	1.031 (1.001–1.061)	0.043	1.057 (1.029–1.086)	<0.001	1.037 (1.002–1.073)	0.039
Creatinine (per 1 µmol/L)	1.002 (1.001–1.003)	<0.001	1.002 (1.001–1.004)	<0.001	1.003 (1.002–1.004)	<0.001	1.003 (1.002–1.005)	<0.001
LVEF (per 1%)	0.959 (0.942–0.977)	<0.001	0.980 (0.963–0.997)	0.020	0.953 (0.933–0.974)	<0.001	0.982 (0.962–1.002)	0.074
Post-PCI TIMI flow grade 3	0.291 (0.190–0.447)	<0.001	0.215 (0.134–0.345)	<0.001	0.247 (0.156–0.392)	<0.001	0.173 (0.103–0.292)	<0.001
Arterial lactate (per 1 mmol/L)	1.167 (1.123–1.212)	<0.001	1.106 (1.055–1.159)	<0.001	1.191 (1.144–1.240)	<0.001	1.121 (1.066–1.180)	<0.001
OHCA	1.697 (1.077–2.672)	0.023	1.021 (0.609–1.712)	0.936	2.023 (1.229–3.329)	0.006	1.018 (0.569–1.822)	0.952
Mechanical ventilation †	5.087 (2.735–9.462)	<0.001	2.808 (1.463–5.391)	0.002	19.98 (4.92–81.21)	<0.001	8.40 (2.02–35.02)	0.003
MCS	1.432 (0.999–2.052)	0.050	1.377 (0.929–2.040)	0.111	1.695 (1.102–2.606)	0.016	1.787 (1.095–2.917)	0.020

Abbreviations: HR = hazard ratio. CI = confidence interval. SBP = systolic blood pressure. LVEF = left ventricular ejection fraction. TIMI = thrombolysis in myocardial infarction. PCI = percutaneous coronary intervention. OHCA = out-of-hospital cardiac arrest. MCS = mechanical circulatory support. † The wide confidence interval for mechanical ventilation in the 30-day analysis reflects the concentration of early deaths among ventilated patients (few 30-day events occurred in non-ventilated patients).

**Table 4:** Attenuation of the serum sodium–mortality association by individual cardiogenic shock severity variables.

**Panel A. Long-Term Mortality**				
**Model**	**Serum Sodium**		**Added Covariate**	
	**HR (95% CI)**	***p***	**HR (95% CI)**	***p***
Sodium alone	1.070 (1.029–1.112)	<0.001	—	—
Sodium + arterial lactate	1.018 (0.977–1.060)	0.405	1.157 (1.109–1.208)	<0.001
Sodium + OHCA	1.061 (1.018–1.106)	0.005	1.329 (0.814–2.172)	0.256
Sodium + mechanical ventilation	1.051 (1.011–1.092)	0.012	4.750 (2.546–8.863)	<0.001
Sodium + IABP-SHOCK II score	1.037 (0.998–1.077)	0.066	1.455 (1.316–1.608)	<0.001
**Panel B. 30-Day Mortality**				
**Model**	**Serum Sodium**		**Added Covariate**	
	**HR (95% CI)**	***p***	**HR (95% CI)**	***p***
Sodium alone	1.087 (1.043–1.132)	<0.001	—	—
Sodium + arterial lactate	1.021 (0.976–1.068)	0.359	1.179 (1.126–1.235)	<0.001
Sodium + OHCA	1.073 (1.027–1.122)	0.002	1.461 (0.844–2.529)	0.175
Sodium + mechanical ventilation	1.063 (1.020–1.107)	0.004	18.03 (4.42–73.44)	<0.001
Sodium + IABP-SHOCK II score	1.045 (1.003–1.088)	0.037	1.607 (1.429–1.806)	<0.001

Abbreviations: HR = hazard ratio. CI = confidence interval. OHCA = out-of-hospital cardiac arrest. IABP-SHOCK II = intra-aortic balloon pump in cardiogenic shock II.

## Data Availability

The data used and/or analyzed in this study are available from the corresponding author upon request.

## Supplementary Materials

The following supporting information can be downloaded at: https://www.mdpi.com/article/10.3390/biomedicines14092086/s1, Table S1: Robustness of the serum sodium–mortality association across alternative model specifications and proportional-hazards diagnostics.

## Abbreviations

AMI	Acute myocardial infarction
AMI-CS	Cardiogenic shock secondary to acute myocardial infarction
BMI	Body mass index
CAD	Coronary artery disease
CCU	Cardiac care unit
CPR	Cardiopulmonary resuscitation
CS	Cardiogenic shock
ECMO	Extracorporeal membrane oxygenation
HF	Heart failure
IABPSHOCK II	Intra-aortic balloon pump in cardiogenic shock II
ICU	Intensive care unit
LVEF	Left ventricular ejection fraction
MAP	Mean arterial pressure
OHCA	Out-of-hospital cardiac arrest
PCI	Percutaneous coronary intervention
PAD	Peripheral artery disease
SBP	Systolic blood pressure
SCAI	Society for Cardiovascular Angiography and Interventions
SGLT2	Sodium glucose cotransporter 2
STEMI	ST-segment elevation myocardial infarction
TIMI	Thrombolysis in myocardial infarction
