# Supplementary material for: Admission Serum Sodium as an Early Integrative Marker of Shock Severity in Acute Myocardial Infarction-Complicated Cardiogenic Shock
Source: Biomedicines. 2026 Sep 16;14(9):2086. doi: 10.3390/biomedicines14092086 (PMC13604549; doi:10.3390/biomedicines14092086)
Supplement: Supplementary file 1 [file biomedicines-14-02086-s001.zip › biomedicines-4549820-supplementary.pdf]

Supplementary Table S1. Robustness of the serum sodium–mortality association across alternative model specifications and proportional-hazards diagnostics.

**Analysis**	**Admission sodium HR (95% CI)**	***P***	**Global PH test P**
Primary model (fully adjusted, long-term)	1.02 (0.98–1.06)	0.46	<0.001§
Time-varying lactate model (lactate effect split at day 30)	1.02 (0.97–1.06)	0.48	0.31
30-day landmark analysis (acute-phase window)	1.02 (0.98–1.07)	0.31	0.20
Model excluding lactate	1.05 (1.01–1.09)	0.02	—
Model including lactate	1.02 (0.98–1.06)	0.48	—

All models are based on the 267 patients with complete covariate data (122 long-term deaths) and adjust for age, systolic blood pressure, heart rate, glucose, creatinine, LVEF, post-PCI TIMI flow grade 3, OHCA, mechanical ventilation, and mechanical circulatory support, with arterial lactate added except where stated. Hazard ratios are per 1 mmol/L increase in admission serum sodium. Across every specification, admission sodium was not independently associated with mortality once arterial lactate was accounted for; the association was significant only when lactate was omitted from the model. § In the primary model the global proportional-hazards (PH) test was violated owing to a time-varying effect of arterial lactate. Allowing lactate a period-specific effect (before vs. after day 30) restored the global PH assumption (P=0.31), and admission sodium itself showed no PH violation (P=0.28); the 30-day landmark model also satisfied the PH assumption (P=0.20). Thus the non-proportionality reflected lactate rather than sodium and did not affect the conclusion.

Abbreviations: HR, hazard ratio; CI, confidence interval; PH, proportional hazards; LVEF, left ventricular ejection fraction; TIMI, Thrombolysis in Myocardial Infarction; PCI, percutaneous coronary intervention; OHCA, out-of-hospital cardiac arrest.
